# Probable Fracture of Base of Skull from a Fall

**Published:** 1856-07

**Authors:** Abraham Livezey

**Affiliations:** Lumberville, Penn.


*Probable Fracture of Base or Scull from a Fall.* By Abraham Livezey, A. M., M. D., Lumberville, Penn.

W. C. B., a farmer, æt. 22, of good habits and constitution, with a fair development of the muscular system, while engaged in painting a dripboard extending across the gable end of a barn, from a ladder at the height of 30 feet, on the 8th of April, 11½ o'clock, A. M., was suddenly precipitated headlong, by the breaking of the ladder, upon a hard knoll of ground; the base of the right parietal bone receiving the shock or force of the fall. He was carried into his father's house, senseless, with a slight epistaxis, and rather a profuse hemorrhage from the right ear.

In less than an hour Dr. Foulke and myself saw him. He was in a muttering, restless unconscious state, with a dull, vacant look, blood constantly oosing from the ear—none now from the anterior nares—skin cold, pallid; pulse slow, feeble and intermittent; no paralysis. We immediately resorted to stimulants internally (which were resisted and swallowed with some apparent difficulty), and externally to friction, sinagisms, &c. After the lapse of five hours, we obtained a slight reaction—sufficient, in the opinion of Dr. F., to warrant a tentative bleeding from the arm—though, in fact, an *anceps remedium*;* and we took six or eight ounces, the pulse not warranting more. An equal amount, we supposed, had been lost by the ear at this time. Twice, during the afternoon, he vomited several ounces of blood. Stimulating and purgative enemata were also resorted to, during the first twelve hours.

On the next day, at 6 o'clock, P. M., he was seen with us by Dr. Neill, Professor of Surgery in the Pennsylvania College of Medicine, whose opinion of the case accorded with ours, previously given, that there was evidently a fracture of the base of the cranium, extending through the petrous portion of the temporal bone; with, probably, a laceration of the substances of the brain. Bladders filled with pounded ice had been applied to his head more or less constantly, from the beginning, and were continued for two weeks, as indicated and as the circumstances of the case would permit. Purgation, by means of calomel, was now resorted to, and grain doses of the same continued every two hours, for a few days, until the evacuations were strikingly characteristic of the action of this mineral. No salivation ensued. The hair was cut close all over his head, and shaved from ear to ear beneath the semi-circular ridge of the occipital bone, for the application of cups and leeches, which were occasionally used. The pulse continued about 50 for some days—then 60 to 65; towards the end of the second week, it varied from 60 to 108 in the twenty-four hours, for two days—at which time the prognosis was grave—but then settled down to 75 or 80. The calomel was followed by the hydrarg. biniod. dissolved in a solution of the potass. iodid., and finally the iodide alone was given for some days. His bladder and rectum were evacuated unconsciously for about a week; after which, from certain manifestations, he was generally placed upon the stool in time. The sinapisms applied to the ankles on the first day, produced, by the end of a week, serious ulcers with profuse discharges. A large inflammatory abscess formed near the patella of the left leg, and discharged freely on the fifteenth day after the accident; another, on the inside of the right thigh, opened a few days after—at which time he was fully sensible of his condition and sufferings when dressings were applied.

But let me revert to the case at the time of the accident, and trace his gradual return to consciousness; and in so doing, the case will present some points of interest to the phrenologist and to the physician believing in a plurality of organs in the brain. I believe "mother" was the only distinct word uttered by the patient for several hours; the next day,

Decidedly.—EDS.

"Sam" (a negro whom he was last with), and the word "whoa," used to check horses, were frequently used; which, together with "gid up," "get along," *&c.*, whilst quiet, and the words "I want," "won't you," "do let," when striving to get up, or out of bed, constituted the chief that was said for three days. He labored for a week or more under the delusion of driving horses. He slept about one-third of the time for the first few days. When wakeful, it required three person to keep him in bed; the restraint, however did not appear to irritate him at all, until after the lapse of a week, when he became furious (excited combativeness?), would strike his attendants, spit in their faces, and use profane language which he was never known to use before. A little later, when restrained, he would look very pitifully and cry.

But I anticipate; after the first few days, he was good-natured, showed a happy disposition, would lie quiet, look about, would spell short words, pronounce the Christian name of a few of his friends, utter the words "go along," and commence whistling or humming a tune, very loudly—a habit he was not much addicted to. This state continued three or four days. Next alternating with this, the organ of destructiveness was excited. He became violent, maniacal, would break his drinking-vessels and anything he could get hold of. Then combativeness seemed to rule, and he would fight, bite, and deal unlucky blows to his attendants when off their guard. At other times he was all love and affection—kissing every person's hand, and not unfrequently would throw his arms around the necks of individuals and caress and kiss them. Then perhaps *cunning* would be the more prominent trait for a time.

Thus passed two weeks and two days, without his apparently knowing a single individual, or calling any one by name—and without heeding a single direct question. The sense of feeling was pretty acute after the first eighteen hours; he was sensitive to the touch and to handling, and would often cry out "*ouch*," particularly when the mustard-blistered surfaces were touched or dressed. The sense of taste was apparently quite defective for a week or more, for he would drink nauseous liquids, oil, or other medicines, alike with water, gruel, or panada. After this time he would eject nauseous medicines with force over his bed or his attendants, indifferently. At the end of a week or ten days, he manifested a great desire for drinks, ice, &c., first noticing drinking vessels, and demanding them thus: "what's that," "let's have it," "bring me some of that," *&c.*, thus expressing his wants, without calling anything by name. He mentioned water only a few times. Names of things or persons were the last to come back to his recollection; and among the first were "a smoke," "tobac," *&c.*, for which he manifested a great desire, though not much addicted to the habit. I should also state, that *alimentiveness*, or desire for food, was very great after the second week—he begged everybody to give him something "nice" to eat.

At the end of two weeks and three days, he was decidedly convalescent—was sitting up warming his feet by the stove; would ask for things, take some interest in books, watches, gold chains, *&c.* He was much concerned about his mother, who at this time was dangerously ill. He rode a distance of nearly a mile on the twentieth day after his injury, to witness the interment of his mother, who died suddenly, and during the funeral ceremony he was, at times, quite affected. From this time his improvement was rapid.—*Boston Med. and Surg. Journ.*

